# Decrease of thyroid function after ischemic stroke is related to stroke severity

**DOI:** 10.1186/s13044-023-00160-w

**Published:** 2023-07-13

**Authors:** Evgeny Sidorov, Aruna Paul, Chao Xu, Claire Delpirou Nouh, Allshine Chen, Albina Gosmanova, Niyaz Gosmanov, David Lee Gordon, Irina Baranskaya, Juliane Chainakul, Robert Hamilton, Alexander Mdzinarishvili

**Affiliations:** 1 Department of Neurology, University of Oklahoma Health Sciences Center, 920 Stanton L. Young Blvd #2040, Oklahoma City, OK 73104 USA; 2 Oklahoma Center for Neurosciences (OCNS), Department of Cell Biology, 940 Stanton L. Young Blvd, BMSB-536, Oklahoma City, OK 73104 USA; 3 Department of Biostatistics and Epidemiology, University of Oklahoma Health Sciences Center, 801 N.E. 13Th Street, Oklahoma City, OK 73104 USA; 4 Department of Medicine, Section of Endocrinology, Diabetes and Metabolism, University of Oklahoma Health Sciences Center, 1000 N. Lincoln Blvd, Oklahoma City, OK 73104 USA; 5 VA Hospital Oklahoma City, 921 NE 13Th Street, 1A-126, Oklahoma City, OK 73104 USA; 6 Department of Psychiatry, 920 Stanton L. Young Boulevard, G. Rainey Williams Pavilion, 3Rd Floor, Oklahoma City, OK 73104 USA

**Keywords:** Thyroid hormones, Acute ischemic stroke, Triiodothyronine, Thyroxin, Stroke severity

## Abstract

**Background:**

Thyroid hormones are of fundamental importance for brain function. While low triiodothyronine levels during acute ischemic stroke (AIS) are associated with worse clinical outcomes, dynamics of thyroid function after AIS remains unknown. Thus, we longitudinally evaluated thyroid hormones after stroke and related them to stroke severity.

**Methods:**

We prospectively traced thyroid stimulating hormone (TSH), free triiodothyronine (fT3), and free thyroxin (fT4) levels from the hyper-acute (within 24 h) to acute (3–5 days) and chronic (3–6 months) stages of ischemic stroke using a mixed regression model. Then, we analyzed whether stroke severity at presentation, expressed by National Institute of Health Stroke Scale (NIHSS), is associated with change in thyroid function.

**Results:**

Forty-five patients were evaluated in hyper-acute and acute stages, while 29 were followed through chronic stage. TSH levels decreased from hyper-acute (2.91 ± 0.65 μIU/mL) to acute (2.86 ± 0.46 μIU/mL) and chronic stages of stroke (1.93 ± 0.35 μIU/m, *p* = 0.95). fT3 levels decreased from hyper-acute (2.79 ± 0.09 pg/ml) to acute (2.37 ± 0.07 pg/ml) stages, but recovered in chronic stage (2.78 ± 0.10 pg/ml, *p* < 0.01). fT4 levels decreased from hyper-acute (1.64 ± 0.14 ng/dl) to acute (1.13 ± 0.03 ng/dl) stages, and increased in the chronic stage (1.16 ± 0.08 ng/dl, *p* = 0.02). One-unit increase in presenting NIHSS was associated with 0.04-unit decrease of fT3 from hyper-acute to the acute stage (*p* < 0.01).

**Conclusion:**

There is a transient decrease of thyroid hormones after ischemic stroke, possibly driven by stroke severity. Larger studies are needed to validate these findings. Correction of thyroid function in acute stroke may be investigated to improve stroke outcomes.

## Introduction

Acute ischemic stroke (AIS) is a major cause of serious disability in the United States [1]. In the last 30 years AIS care significantly improved due to increasing utilization of IV thrombolysis and endovascular thrombectomy. However, these methods are only effective during short period after beginning of stroke symptoms and may be inaccessible for many patients [2]. Therefore, there is a need to find alternative strategies to improve stroke outcomes.

Thyroid hormones play crucial role in brain maturation, function, and may be essential in stroke pathophysiology [3]. Several human studies, including one meta-analysis, suggested that patients with low triiodothyronine levels during acute stroke have more severe impairment and worse mortality [4–6]. Animal experiments, on the other hand, showed that supplementation of thyroid hormones to a mouse with transient middle cerebral artery occlusion decreases the size of infarction and improves neurological outcomes [7]. Thus, supplementation of thyroid hormones during AIS may be beneficial and should be investigated. However, before initiating such study, it is important to understand the dynamics of thyroid function after stroke, and identify possible changes in thyroid hormones driven by acute cerebral ischemia [7, 8]. Understanding these changes will help to plan timing and amount of supplemented thyroid hormones. To our knowledge, though, most clinical studies evaluated thyroid hormones at single point in time, days after AIS, and did not trace them from the beginning of stroke symptoms. Therefore, we performed pilot investigation of thyroid function from first hours, to days, and months after ischemic stroke. In addition, we evaluated whether change in thyroid function is associated with AIS severity at presentation.

## Materials and methods

### Study design and participants

**Fig. 1:**
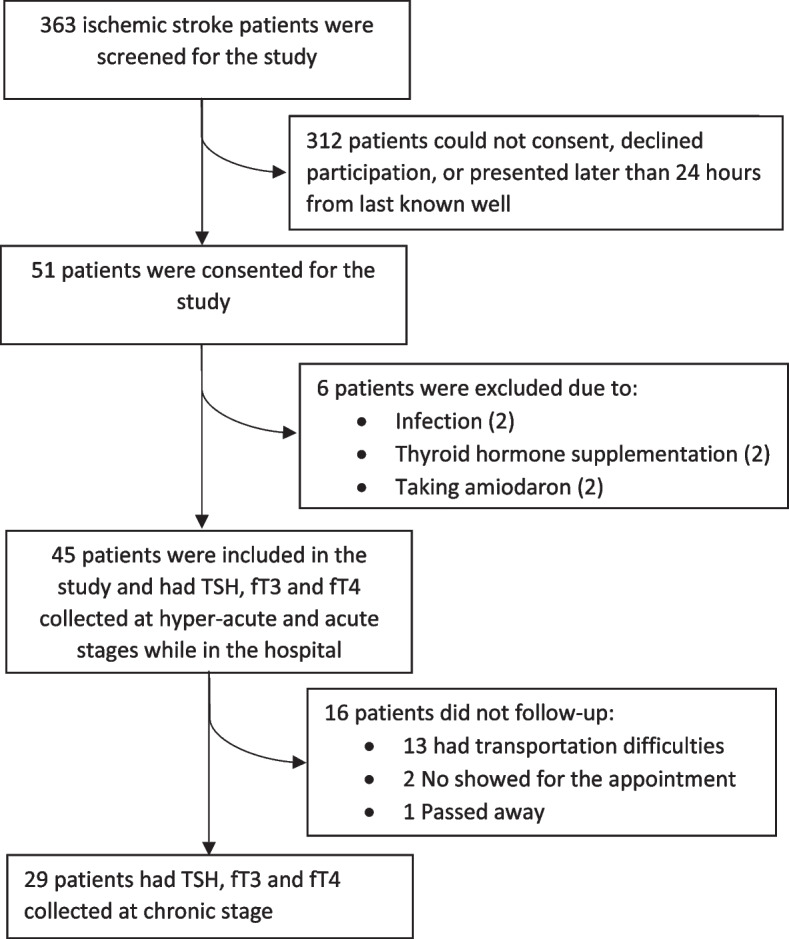
Diagram of study enrollment from 2016 to 2018

Fifty-one nonconsecutive AIS patients were consented for this study, six met exclusion criteria (Fig. 1), and forty-five were included in the study. This study was approved by the local institutional review board. We evaluated thyroid function in a longitudinal fashion, collecting serum TSH, fT3, and fT4 in the hyper-acute (within 24 h of patient’s last known well), acute (2–5 days after admission), and chronic (3–6 months) stages of ischemic stroke. As previous studies indicated that metabolic markers altered in ischemic stroke reach normal levels by 90 days, chronic stage values may represent baseline thyroid function [9, 10]. TSH was measured by immunoradiometric assay and fT3, fT4 were measured by radioimmunoassay in the local laboratory. Normal ranges are as follows: TSH (0.35—4.94 μIU/mL); fT3 (1.71–3.71 pg/ml); fT4 (0.7–1.5 ng/dL).

Study was performed from 2016 to 2018 at Comprehensive Stroke Center, each patient presented as “stroke alert”, a process in which acute stroke patients are evaluated for thrombolysis and endovascular thrombectomy, and initiated within 24 h of time last known well. Hyper-acute and acute stage blood samples were collected during inpatient stay, while chronic stage samples were collected during follow-up clinic visit. We collected patients demographic (age, gender, ethnicity), and clinical (hypertension, hyperlipidemia, diabetes mellitus, smoking, previous history of AIS, atrial fibrillation, IV thrombolysis, and endovascular thrombectomy status) information. For ischemic stroke definition, patients underwent diffusion brain MRI within 48 h of hospital admission. All patients received computed tomography angiogram. We used the National Institutes of Health Stroke Scale (NIHSS; 0–42 points reflecting none and the most severe neurological deficit) to assess stroke severity at presentation [11]. During follow-up visit, we also reviewed patients’ charts to assess for recurrent stroke.

We excluded patients with: (1) hemorrhagic conversion of ischemic stroke and formation of parenchymal hematoma defined by Heidelberg bleeding classification [12]; (2) systemic infection at presentation or during admission with fever > 38.0 °C, elevated white blood cells, diagnosis of pneumonia, or urinary tract infection; (3) recurrent stroke in chronic stage; and (4) chronic thyroid problems, including patients receiving thyroid hormone supplementation, antithyroid medications, corticosteroids, lithium, metformin and amiodarone.

### Statistical analysis

The clinical and demographic variables were summarized using means for continuous variables, while the numbers and percentages were reported for categorical variables. We used mixed regression model to evaluate change in thyroid function (TSH, fT3, fT4) from hyper-acute, to acute and chronic stages of ischemic stroke. In addition to age, ethnicity, hypertension, diabetes, smoking and body mass index (BMI), we used presenting NIHSS as a covariate to evaluate whether stroke severity is associated with change of TSH, fT3, and fT4. Average values of TSH, fT3, fT4 and NIHSS are reported with standard error. All analyses were performed using SAS version 9.4 M5. Statistical significance was assessed with a *p*-value of 0.05.

## Results

We performed thyroid function tests on all 45 patients in the hyper-acute and acute stages of stroke, while in chronic stage we analyzed data only on 29 patients. Out of 16 patients lost to follow-up, 13 have chosen to follow at local office because of transportation problems, 2 patients did not show up for the appointment, 1 was deceased***.*** Demographic and clinical characteristics are presented in Table 1. Most of the patients had strokes in the middle cerebral artery territory (80%), embolism was the most common cause of stroke (50.9%). The average time of serum collection from last time known well was: in hyper-acute stage 5.68 ± 0.21 h, in acute stage 2.58 ± 0.39 days, and in chronic stage 115.92 ± 0.34 days. Average presenting NIHSS was 10.08 ± 0.82, and average infarction volume was 28 ± 0.17. Lost to follow-up patients had similar clinical and demographic characteristics to those who completed the study.

**Table 1:** Demographic and clinical characteristics of the study cohort (*N* = 45). Mean and standard error are reported for continuous variables, while percentages are reported for categorical variables

Age (years)	62.27 ± 2.02
Gender (female)	27 (60%)
Ethnicity: African American	8 (17.8%)
White	37 (82.2%)
Hypertension	32 (71.1%)
Hyperlipidemia	22 (48.9%)
Diabetes mellitus	14 (31.1%)
Smoking	24 (53.3%)
Previous history of stroke	6 (13.3%)
Atrial fibrillation	11(23%)
Average NIHSS	10.07 ± 0.77
Infarction volume (ml)	28 ± 0.25
Thrombolysis	30 (66.7%)
Endovascular thrombectomy	15 (33.3%)

**Fig. 2:**
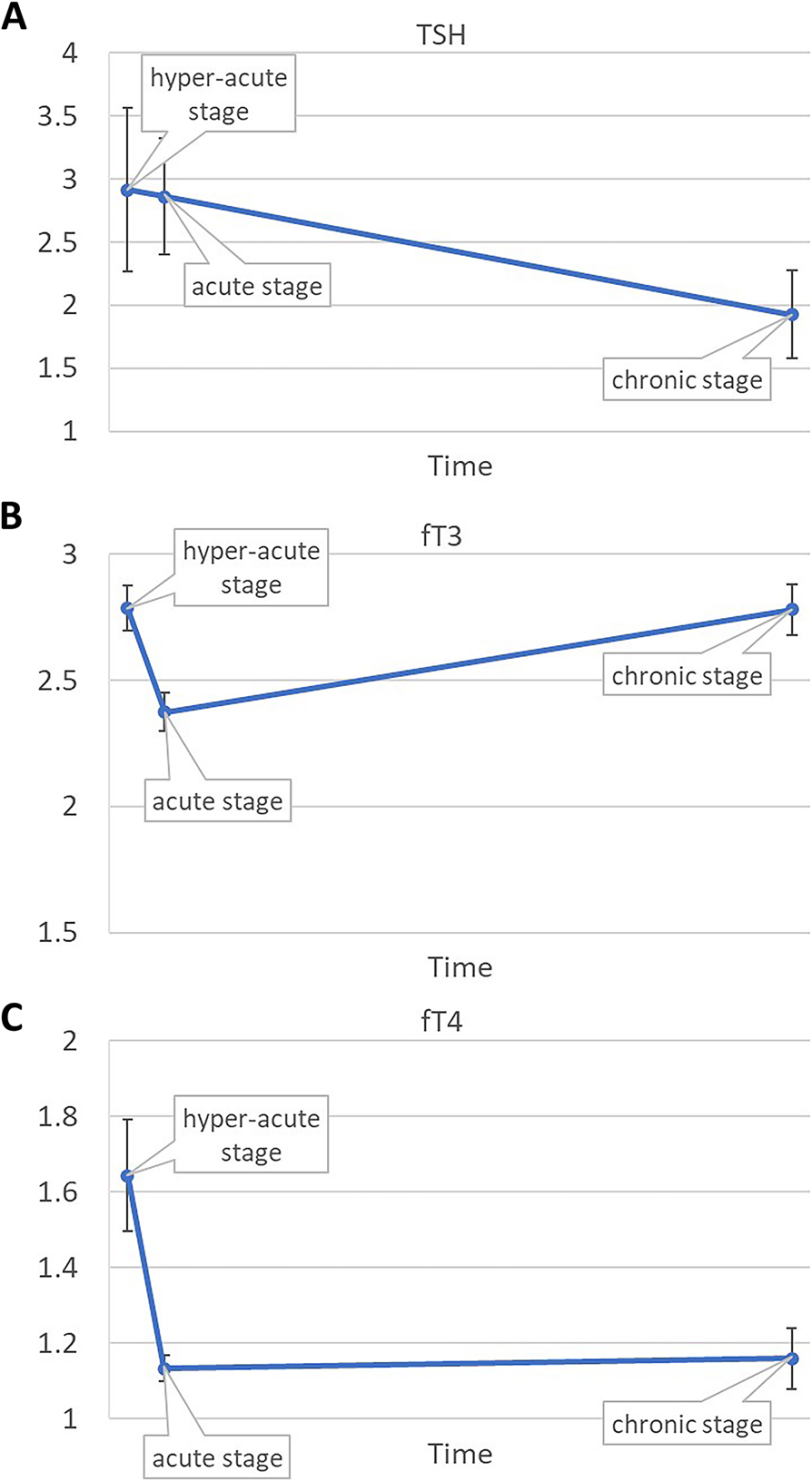
(**A**) TSH levels decrease from hyper-acute to acute and chronic stages of stroke; (**B**) fT3 levels decrease from hyper-acute stage to acute stage, and then increase in chronic stage of stroke; (**C**) fT4 levels decrease from hyper-acute stage to acute stage, and increase in the chronic stage of stroke

Mixed regression analysis of TSH showed decline from hyper-acute (2.91 ± 0.65 μIU/mL) to acute (2.86 ± 0.46 μIU/mL) and to chronic stages (1.93 ± 0.35 μIU/mL) of stroke (*p* = 0.95, Fig. 2A). Mixed regression analysis of fT3 showed decline from hyper-acute (2.79 ± 0.09 pg/ml) to acute (2.37 ± 0.07 pg/ml) stage, and increase in chronic stage of stroke (2.78 ± 0.10 pg/ml) (*p* < 0.01, Fig. 2B). Mixed regression analysis of fT4 showed decline from hyper-acute (1.64 ± 0.14 ng/dl) to acute (1.13 ± 0.03 ng/dl) stage, and then increase in chronic stage of stroke (1.16 ± 0.08 ng/dl) (*p* = 0.02, Fig. 2C). Presenting NIHSS associated with decline of fT3 *(β* = -0.04; *p* < *0.01*) from hyper-acute to acute stage, but was not associated with change of TSH (*β* = 0.07; *p* = 0.14) or fT4 (*β* = -0.01; *p* = *0.09*) (Table 2). One-unit increase in presenting NIHSS was associated with 0.04-unit decrease of fT3 from hyper-acute to acute stage.

**Table 2:** Mixed regression analysis of TSH, fT3, and fT4. Presenting National Institute of Health Stroke Scale (NIHSS), age, ethnicity, hypertension, diabetes, smoking and body mass index (BMI) are covariates. β: regression coefficient

	TSH		fT3		fT4	
	β	*p*-value	β	*p*-value	β	*p*-value
Presenting NIHSS	0.06	0.54	-0.03	0.02	-0.01	0.38
AGE	0.03	0.43	-0.01	0.06	0.01	0.31
Ethnicity	-1.65	0.17	-0.31	0.05	0.05	0.64
Hypertension	-0.36	0.71	-0.11	0.38	-0.02	0.86
Diabetes mellitus	3.54	< 0.01	-0.06	0.64	0.03	0.75
Smoking	0.2	0.84	-0.02	0.84	-0.10	0.24
BMI	0.09	0.21	0.01	0.50	0.01	0.35
Change from hyper-acute to acute stage of stroke	-0.06	0.93	-0.35	< 0.01	-0.47	0.01
Change from acute to chronic stage of stroke	-0.19	0.77	-0.04	0.80	-0.45	0.02

## Discussion

Our pilot investigation shows how thyroid function changes after AIS, and identifies stroke severity as factor associated with decrease of fT3. Essentially, thyroid hormones decrease shortly after beginning of stroke symptoms, bottom during first days, and, then, recover in chronic stage, while TSH continues to decrease. More severe strokes probably lead to stronger decrease of fT3, which eventually results in worse clinical outcomes and mortality, as was reported by other investigators [4–6].

We have two possible explanations of observed thyroid function change after stroke. First is a suppression of hypothalamic-pituitary-thyroid axis from release of corticosteroids in response to stroke-induced body stress, which leads to decrease of TSH production by pituitary gland and corresponding decrease of fT3 and fT4 [13]. Similar response was previously described in patients with myocardial infarction, a disease with pathophysiology similar to ischemic stroke, which also puts significant stress on the body [14]. Second mechanism may be related to a decrease of deiodinase activity, an enzyme, which converts thyroxin to triiodothyronine [15]. Previous studies indicated that decreased deiodinase activity might be caused by variety of acute illnesses, including sepsis and cardiovascular collapse, leading to decline in thyroid hormones during the first 24 h [16]. One study correlated decrease of deiodinase activity and triiodothyronine levels with severity of traumatic brain injury, which corresponds to our findings of decreased fT3 in patients with more severe strokes [17]. During recovery time, acute-phase reactions subside, patients improve their functional status, and thyroid hormones gradually recover. A continuous decline in TSH may reflect development of central hypothyroidism, but in our study, decrease of TSH was not statistically significant.

In previous studies, low triiodothyronine levels were associated with worse outcomes and mortality after AIS, however, cause-effect of such relationship remained unknown [4–6]. Authors speculated that lower triiodothyronine may decrease astrocytic glutamate uptake and promote cytotoxic edema, which eventually worsened impairment after stroke[7, 18]. Our study adds to the results of previous investigations. It shows that decrease of fT3 levels is driven by stroke severity, therefore, worse AIS outcomes and mortality associated with low fT3 levels in previous studies may reflect the relationship between AIS severity and fT3 decrease identified in our study. On the other hand, if low triiodothyronine promotes brain damage, it may create a vicious cycle where more severe strokes lead to decrease of triiodothyronine, and then, lower triiodothyronine farther facilitates brain damage through described above mechanism. In this view, correction of triiodothyronine from first hours to months after stroke may have a potential to improve outcomes.

The strengths of our study include a well-characterized patient cohort with available longitudinal data and specimens collected in the hyper-acute, acute, and chronic stages of stroke. Limitations include comparatively small sample size, low follow-up rate, and administration of iodine-based contrast to all patients, which could affect study result. Given a pilot nature of the study, results should be interpreted with caution as they require verification in larger trials.

## Summary/conclusion

Overall, our findings demonstrate that thyroid function decreases shortly after acute ischemic stroke, and that decrease of fT3 is associated with stroke severity. Recovery after ischemic stroke is accompanied by an increase in thyroid hormone levels. If results of our study are replicated on a larger cohort, correction of triiodothyronine levels may be further investigated to improve stroke outcomes.
